# Premium Intraocular Lenses in Glaucoma—A Systematic Review

**DOI:** 10.3390/bioengineering10090993

**Published:** 2023-08-22

**Authors:** Ashley Shuen Ying Hong, Bryan Chin Hou Ang, Emily Dorairaj, Syril Dorairaj

**Affiliations:** 1 Yong Loo Lin School of Medicine, National University of Singapore, Singapore 119228, Singapore; hongashley@gmail.com; 2 Department of Ophthalmology, National Healthcare Group Eye Institute, Tan Tock Seng Hospital, Singapore 308433, Singapore; 3 Department of Ophthalmology, National Healthcare Group Eye Institute, Woodlands Health Campus, Singapore 768024, Singapore; 4 Charles E. Schmidt College of Medicine, Florida Atlantic University, Boca Raton, FL 33431, USA; emilyadorairaj@gmail.com; 5 Department of Ophthalmology, Mayo Clinic, Jacksonville, FL 32224, USA; dorairaj.syril@mayo.edu

**Keywords:** premium intraocular lens, glaucoma, multifocal intraocular lens, extended depth of focus intraocular lens, toric intraocular lens

## Abstract

The incidence of both cataract and glaucoma is increasing globally. With increasing patient expectation and improved technology, premium intraocular lenses (IOLs), including presbyopia-correcting and toric IOLs, are being increasingly implanted today. However, concerns remain regarding the use of premium IOLs, particularly presbyopia-correcting IOLs, in eyes with glaucoma. This systematic review evaluates the use of premium IOLs in glaucoma. A comprehensive search of the MEDLINE database was performed from inception until 1 June 2023. Initial search yielded 1404 records, of which 12 were included in the final review of post-operative outcomes. Studies demonstrated high spectacle independence for distance and good patient satisfaction in glaucomatous eyes, with positive outcomes also in post-operative visual acuity, residual astigmatism, and contrast sensitivity. Considerations in patient selection include anatomical and functional factors, such as the type and severity of glaucomatous visual field defects, glaucoma subtype, presence of ocular surface disease, ocular changes after glaucoma surgery, and the reliability of disease monitoring, all of which may be affected by, or influence, the outcomes of premium IOL implantation in glaucoma patients. Regular reviews on this topic are needed in order to keep up with the rapid advancements in IOL technology and glaucoma surgical treatments.

## 1. Introduction

Premium intraocular lenses (IOLs) are broadly considered to include presbyopia-correcting IOLs (multifocal IOLs (MFIOLs), extended depth of focus (EDOF) IOLs, accommodative IOLs), and toric IOLs for astigmatism correction. Compared with traditional monofocal IOLs, premium IOLs offer the benefit of better unaided visual acuity, greater spectacle independence, and higher patient satisfaction. In recent years, significant technological advances have been made in cataract surgery and IOL technology, resulting in more precise and predictable refractive outcomes [1]. Due to increasing patient expectation and demand for spectacle independence, premium IOLs have been increasingly adopted in clinical practice in recent years [2]. Global revenue from premium IOL usage is expected to grow at a compound annual growth rate (CAGR) of 9.03% from USD 1.5 billion in 2021 to USD 2.5 billion in 2028, compared to a 6.2% CAGR revenue growth for traditional IOLs [3].

While cataract is the most prevalent cause of reversible loss of vision, glaucoma remains the leading cause of irreversible blindness, characterized by a progressive optic neuropathy with degeneration of retinal ganglion cells and visual field loss [4]. It is estimated that one in five people undergoing cataract surgery have glaucoma or ocular hypertension, with the incidence of both cataract and glaucoma increasing with age [5]. Despite their benefits, many surgeons traditionally exercise caution in implanting premium IOLs, particularly presbyopia-correcting lenses, in patients with glaucoma. Concerns arise regarding contrast sensitivity (CS) loss and subjective visual disturbances such as glares and haloes, which may be more debilitating in patients with glaucomatous visual loss. Pathological changes in glaucoma may also potentially interact with the optical effects of MFIOLs [6]. However, advancements in premium IOL technology and development are enabling improved visual and refractive outcomes, with reduced side effects and less compromise to contrast sensitivity. More studies have also begun to report outcomes of premium IOLs in eyes with glaucoma and associated glaucomatous visual field loss.

However, there has not been a recent systematic review of literature regarding the use of premium IOLs in glaucoma patients, with the last extensive review published more than a decade ago [6]. This systemic review aims to summarize the current available literature reporting the surgical outcomes of premium IOL implantation in eyes with glaucoma. Pre-operative considerations and patient selection factors will also be discussed.

### 1.1. Overview of IOL Types

#### 1.1.1. Monofocal IOLs

Monofocal IOLs provide excellent outcomes for distant vision, with the benefit of generally low cost and low frequency of photic phenomena such as glares and haloes [7]. They are the safest IOL choice for patients with pre-existing ocular pathology as they do not split light. However, as they only provide one focus point, they fail to deliver spectacle independence for near and intermediate vision. 

Monovision is a surgical option correcting distant vision in the dominant eye while the non-dominant eye is corrected for near or intermediate vision, relying on neural adaptation to achieve a broader range of functional vision [8]. Generally utilizing monofocal IOLs, this option has proven a cost-effective method to provide spectacle independence, while avoiding the photic adverse effect caused by MFIOLs. It is best suited for patients prioritising spectacle independence. However, it is also associated with loss of depth perception and suboptimal vision at intermediate distances [8].

#### 1.1.2. Multifocal IOLs (MFIOLs) 

MFIOLs, first approved by the United States Food and Drug Administration (US FDA) in 1997 [9], come in varying optical designs, such as diffractive, refractive, bifocal, trifocal, or hybrid IOLs, and provide multiple focal points. Refractive MFIOLs, such as the Mplus (Oculentis, GmbH, Berlin, Germany), achieves multifocality via light refraction based on Snell’s law [10]. Their design is rotationally symmetrical, with two or more concentric rings of different curvature radii and optical power on the front surface of the lens [11]. However, the lens performance is influenced by IOL centration as this affects the light percentage passing through the various optical zones [11]. Additionally, near visual acuity (VA) also depends on pupil size due to the near focus zone of the MFIOL being concentrically allocated [12].

Diffractive MFIOLs, such as the Acrysof ReSTOR (Alcon Laboratories, Fort Worth, TX, USA) and the Tecnis (Abbott Medical Optics, Johnson & Johnson Vision, Santa Ana, CA, USA), achieve multifocality via light interference based on the Huygens–Fresnel principle [13]. They feature multiple concentric rings with diffractive micro-structures separated by steps 2 μm in height that work independently regardless of pupil size, creating diffractive wave patterns that can focalize light rays on two or more foci. However, as light rays pass through multiple diffractive surfaces, this causes energy loss and reduces contrast sensitivity and increases the frequency of glares and haloes in patients [14].

While both diffractive and refractive MFIOLs produce similar uncorrected visual acuity, diffractive MFIOLs have been observed to provide better unaided near visual acuity [15,16]. Furthermore, refractive MFIOLs tend to cause more halo or glare symptoms due to light scattering at the transitional zone between the distant and near focus of the MFIOL [17]. Various studies [14,15,16,17,18] have shown that compared to traditional monofocal IOLs, MFIOLs offer greater spectacle independence but have a higher risk of glare, halo, and lower contrast sensitivity [19,20]. In patients with MFIOLs, more than a third of patients may report increased glares and haloes [21], and multiple meta-analyses [20] have reported a decrease in CS. However, some studies [22] continue to produce conflicting results. There are also several contraindications to MFIOL implantation, such as corneal aberrations, asymmetric capsulorrhexis, haptics deformation, or lens subluxation, all of which can lead to IOL decentration, an increase in higher order aberrations, and diminished object contrast discrimination [10].

#### 1.1.3. Extended Depth of Focus (EDOF)

The Extended Depth of Focus (EDOF) technology, applied in IOLs such as the Tecnis Symfony (Johnson & Johnson Vision, Santa Ana, CA, US), was first approved by the US FDA in 2016. This recent innovation creates a single elongated focal point to enhance depth of focus and range of vision [21], effectively providing satisfactory near and intermediate vision while addressing limitations of MFIOLs, including negative photic phenomena such as glares and haloes [23,24]. Higher order aspheric monofocal IOLs, which are designed to provide improved intermediate vision, achieve this by redistributing power from the periphery to the centre of the IOL, enhancing depth of focus [25,26]. The structure of EDOF IOLs is based on a diffractive echelette design forming a step structure to achieve constructive interference of light from different lens zones to produce a novel light diffraction pattern [27]. Image quality is further enhanced through proprietary achromatic technology and negative spherical aberration correction [28]. Since 2016, several types of EDOF IOLs have been made commercially available, including the Mini WELL (Sifi Medtech, Catania, Italy), IC-8 (AcuFocus Inc., Irvine, CA, USA) and Wichterle Intraocular Lens-Continuous Focus (Medicem, Kamenné Zehrovice, Czech Republic).

EDOF IOLs enhance correction of chromatic aberration and maintain good CS that may be comparable to that of monofocal IOLs [21,29,30]. In terms of visual outcomes, EDOF IOLs have demonstrated better near [27] and intermediate vision [31] compared to monofocal IOLs, but worse outcomes than trifocal IOLs. However, there have been conflicting results regarding CS outcomes following EDOF implantation. Certain studies [27] have demonstrated a decrease in CS in eyes with EDOF IOLs under scotopic conditions, compared to eyes with monofocal IOLs. However, Pedrotti et al. [29] reported no significant difference, while Mencucci et al. [32] reported that EDOF IOLs performed significantly better than trifocal IOLs under both photopic and scotopic conditions. 

#### 1.1.4. Accommodative IOLs

Accommodative IOLs aim to preserve the ocular dioptric system accommodation capacity that is lost after cataract extraction [14]. These are dynamic IOLs that act independently of pupil size, creating a pseudo-accommodative phenomenon via anterior displacement of the lens optic plate, increasing the dioptric power of the eye and improving spectacle independence [33]. The design structure includes single optic, dual optic, and curvature change IOLs. They provide good far and intermediate vision as well as CS but are limited at near visual acuity and have post-operative outcome variability, necessitating further correction for near vision, and they may also have a higher risk of capsular contraction and opacification [34]. 

A recent review by Ong et al. [35] showed that accommodative IOLs had better near visual acuity at 6 months compared to monofocal IOLs but had greater posterior capsular opacification affecting distance visual acuity. Compared to MFIOLs, the optical plate in accommodative IOLs maintains the same power in every point without any transition areas, resulting in decreased adverse photic phenomenon such as glares, halos, blurs, and glows [17,36,37].

Accommodative IOL are advantageous for glaucoma patients as these lenses do not decrease CS [34]. However, they have increased risk of capsular contraction, commonly seen in pseudo-exfoliation patients, a condition associated with weakened zonules, further decreasing functionality of IOL accommodative system [34].

#### 1.1.5. Toric IOLs

Toric IOLs are astigmatism-correcting lenses that allow for a specific focal point. Zvornicanin et al. [2] conducted a review of recent trials utilizing premium IOL in eyes with cataract without any other ocular pathology and demonstrated a UDVA of 0.3 logMAR in 70–95% of patients, with a residual astigmatism of 1 D or less noted in 67–88% of patients, and spectacle independence reported in 60–85% of patients.

## 2. Materials and Methods

### 2.1. Search Strategy

A literature search was performed in MEDLINE bibliographic database from inception to 1 June 2023. The following key terms were utilized in combination: “multifocal*”, “bifocal*”, “trifocal*”, “diffractive*”, “EDOF”, “extended depth of focus”. The detailed search strategy is available in Supplementary Material (Table S1). References of sources and previous reviews were hand-searched to identify additional relevant articles. Articles were viewed through Rayyan (Qatar Computing Research Institute, Doha, Qatar) and duplicates were identified and removed.

### 2.2. Study Selection

Studies which reported surgical outcomes of premium IOL implantation in glaucoma eyes were shortlisted for data extraction. The article sieve was conducted by two independent reviewers (ASYH, ED), and each article was reviewed by both reviewers who were blinded to each other’s decisions. Disputes were resolved through consensus discussion between the reviewers, followed by arbitration from a third reviewer if necessary. The inclusion and exclusion criteria are listed in Table 1.

### 2.3. Data Extraction 

For each included trial, two reviewers (ASYH, ED) extracted data at the longest point of follow up, abstracted them into an Excel spreadsheet (Microsoft Corp, Albuquerque, NM, USA), and checked for conflicting data entries. Differences were discussed and resolved with a third reviewer where necessary. 

Data extracted included study characteristics, baseline patient information (age, gender, type of pathology and surgery), baseline visual field parameters, Pre-operative intraocular pressure, and number of glaucoma medications. For continuous variables, mean and standard deviation were abstracted. For categorical variables, frequency and percentages were abstracted. 

All outcomes pertained to surgical results following premium IOL implantation in eyes with glaucoma. Primary outcomes included uncorrected and corrected distance and near visual acuity. Secondary outcomes included spectacle independence, photic phenomena (glares and haloes), astigmatism, contrast sensitivity, and patient satisfaction. 

### 2.4. Quality and Risk of Bias Assessment 

Assessments on the risk of bias and certainty of evidence was performed on the final list of studies included in our review. Risk of bias was ascertained at the study level and assessed by 2 reviewers independently and in duplicate. Conflicts were resolved by consensus, with arbitration by a third reviewer if necessary. 

An assessment of the methodology quality of the cohort studies was performed using the domains of the Newcastle–Ottawa Scale [38], considering (1) the selection of cohorts; (2) the comparability of cohorts; and (3) the assessment of outcomes. Studies with <5 stars are considered low quality, 5–7 stars moderate quality, and >7 stars high quality. Non-controlled trials (such as case reports and case series) included in this study were assessed using the modified Newcastle–Ottawa scale [39] based on four domains: selection; ascertainment; causality; and reporting.

## 3. Results

**Figure 1:**
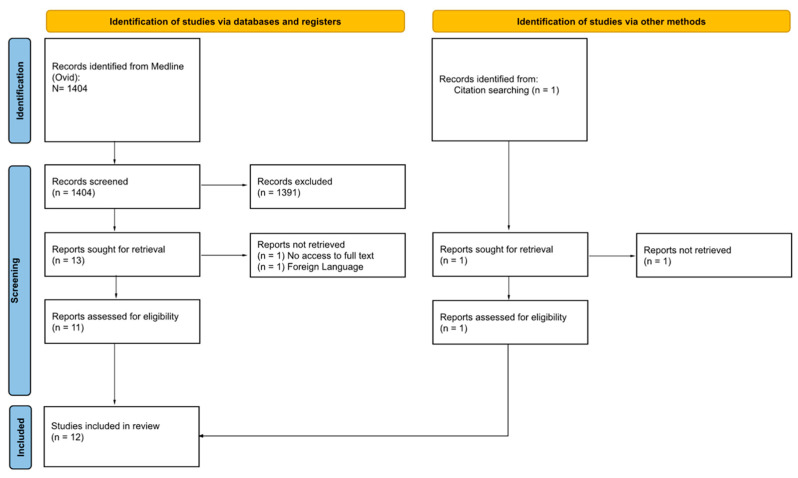
Preferred reporting items for systematic reviews and meta analyses (PRISMA) flow diagram.

Our search yielded 1404 records in total. After screening based on title and abstract, 1391 references were excluded. Full-text assessment was performed on the remaining 13 records; 2 were not retrieved, and 1 additional study was included from citation searching (Figure 1). Twelve studies [22,40,41,42,43,44,45,46,47,48,49,50] fulfilled the inclusion criteria. 

### 3.1. Methodology Quality and Risk of Bias

The results of the risk of bias assessment conducted using the Newcastle–Ottawa Scale [1] for cohort studies and modified Newcastle–Ottawa scale [2] for case results are shown below (Table 2 and Table 3).

All eight cohort studies [3,4,5,6,7,8,9,10] had high NOS ranks and the mean NOS score was 7.625. Three studies lost points because there was absence of a control group with non-glaucomatous eyes. The findings of both reviewers were similar, regardless of whether the material appeared biased.

1. *Selection*: (Question 1) Does/Do the patient(s) represent(s) the whole experience of the investigator (center), or is the selection method unclear to the extent that other patients with similar presentations may not have been reported?
2. *Ascertainment:* (Question 2) Was the exposure adequately ascertained? (Question 3) Was the outcome adequately ascertained?
3. *Causality:* (Question 4) Were other alternative causes that may explain the observation ruled out? (Question 5) Was there a challenge/rechallenge phenomenon? (Question 6) Was there a dose-response effect? (Question 7) Was follow-up long enough for outcomes to occur?
4. *Reporting:* (Question 8) Is/Are the case(s) described in sufficient detail to allow other investigators to replicate the research or to allow practitioners to make inferences related to their own practice?

### 3.2. Patient Characteristics 

The qualitative analysis included a pooled total of 399 glaucomatous eyes from 12 studies. The mean age was 73.8 years old, with a male-to-female ratio of 19:20. Three studies originated from the USA, four studies from Japan, three studies from Spain, one study from Australia, and one study from the UK. Three studies [40,41,49,50] reported on EDOF IOL outcomes, six studies [22,42,44,45,46,47] reported on toric IOL outcomes, one study [48] reported on MFIOL outcomes, and one study [43] reported outcomes from EDOF, bifocal, and trifocal IOL implantation. Study characteristics (author, publication year, sample size, age range, IOL type, surgery type, and outcomes) were extracted and summarized in Tables S2–S4 (Supplementary Material).

### 3.3. Surgical Outcomes from Trials 

#### 3.3.1. Spectacle Independence 

Five studies [22,40,43,49,50] reported on spectacle independence in glaucomatous eyes. Ferguson et al. [40] implanted 52 eyes with mild open angle glaucoma with EDOF non-toric or toric IOLs (AcrySof IQ Vivity/AcrySof IQ Vivity Toric, Alcon Laboratories, Fort Worth, TX, USA) and demonstrated a high rate of spectacle independence post-operatively in both toric and non-toric IOL groups (spectacle independence rates: 92% for distance tasks; 50% for intermediate tasks; and 38% for near tasks). Ouchi et al. [22] implanted 15 eyes (11 patients) with coexisting ocular pathologies (including 4 glaucoma eyes—1 with acute angle closure glaucoma; 3 with normal tension glaucoma) with MFIOLs (LENTIS MPlus LS-313MF30 and the LENTIS Mplus Toric LU-313MFT (Oculentis GmbH, Berlin, Germany)). All patients were completely spectacle independent for distance vision. For distance vision, 11 patients (100%) rated their quality of vision as 4 or higher (very good or good) among 5 items, and 7 patients (64%) rated it as 5 (very good). For near vision, the results of the glaucoma patients were not individually shared and thus not reported in this review. Of note, however, is that Ouchi et al. included only a small sample size of glaucoma eyes (4/15 eyes with coexisting ocular pathology). Sanchez-Sanchez et al. [43] implanted bifocal (AcrySof ReSTOR +3.00, Alcon Laboratories, Fort Worth, TX, USA) and trifocal (AcrySof Panoptix, Alcon Laboratories, Fort Worth, TX, USA, Fine Vision PhysIOL, Liege, Belgium) IOLs in nine patients with glaucoma (77.8% Bifocal Lens; 22.2% Trifocal Lens) and in nine patients with pre-perimetric glaucoma (77.8% Bifocal Lens; 22.2% Trifocal Lens). A total of 68% of patients achieved spectacle independence for distance tasks, 89% for intermediate tasks, and 56% for near tasks. Rementeria-Capelo et al. [49] evaluated visual outcomes in 25 control patients and 25 study patients with ocular pathology. Study patients included six patients with glaucoma and two with ocular hypertension undergoing bilateral combined iStent and cataract surgery with EDOF IOL (AcrySof IQ Vivity; Alcon Laboratories, Fort Worth, TX, USA). All study patients were spectacle independent for distance. For near vision, the results of the glaucoma patients were not individually shared and thus not reported in this review. Of note, however, is that Rementería-Capelo et al. [49] included only a small sample size of glaucoma eyes (8/25 study patients). Kerr et al. [50] implanted an EDOF IOL (AcrySof IQ Vivity; Alcon Laboratories, Fort Worth, TX, USA) in 32 glaucomatous eyes (29 with primary open angle glaucoma; 3 with secondary open-angle glaucoma) and a monofocal IOL (Clareon/SN6Atx/SN60WF; Alcon Laboratories, Fort Worth, TX, USA) in 26 glaucomatous eyes (23 with primary open angle glaucoma; 3 with secondary open angle glaucoma), with a trans-trabecular micro-bypass stent (iStent; Glaukos Corp., San Clemente, CA, USA)) or Schlemm canal microstent (Hydrus Microstent; Ivantis, USA) concurrently implanted in 14 eyes in the EDOF group. In the EDOF group, spectacle independence was high, with 13 patients never requiring spectacles, 3 patients rarely requiring spectacles for distance and intermediate activities, and 7 patients never requiring spectacles for near activities. Spectacle independence for intermediate and near activities was significantly better in the EDOF group compared to the monofocal group; the number of patients in the monofocal group always requiring spectacles for intermediate and near activities was 4 and 11, respectively. 

In summary, five studies reported high spectacle independence rates for distance vision. These results were consistent across different IOL types (including bifocal, trifocal, and toric IOLs) and observed also in studies examining outcomes of premium IOL implantation in combination with glaucoma surgery. For near vision, specific outcomes for glaucomatous eyes were not reported in two out of the five studies. In the remaining three studies, the results varied for near vision.

#### 3.3.2. Contrast Sensitivity

Five studies [22,40,41,43,49] reported CS outcomes following premium IOL implantation in glaucomatous eyes. Sanchez-Sanchez et al. [43] reported that patients with glaucoma implanted with MFIOLs had poorer monocular visual acuity than healthy controls and lower contrast sensitivity values at high spatial frequencies—at 12 cycles per degree, binocular CS values for healthy, glaucoma, and pre-perimetric glaucoma eyes were 2.11, 1.87, and 2.05, respectively. However, there was no clinically significant difference in CS between patients with pre-perimetric glaucoma and healthy controls. Ouchi et al. [49] demonstrated that even after MFIOL implantation, CS in all eyes with various ocular pathologies including glaucoma patients (a prior history of acute glaucoma, NTG) were still comparable to those of normal healthy subjects. Ferguson et al. [40] showed favorable CS results following EDOF IOL implantation: mean binocular mesopic CS achieved was 1.76 ± 0.16 at a spatial frequency of 1 cycle-per-degree (cpd). Bissen-Miyajima et al. [41] evaluated outcomes of diffractive EDOF IOLs (Symfony^®^, models ZXR00V and ZXV150-375, Johnson and Johnson Surgical Vision, Santa Ana, CA, USA) in 16 NTG eyes and demonstrated that their post-operative visual function was mostly comparable to those of normal eyes following implantation of the same IOLs, with post-operative CS within the normal range, except for four eyes at 18 cycles per degree. Rementeria-Capelo et al. [49] showed no difference in CS after EDOF IOL implantation in all patients in both control and study groups with glaucoma and ocular hypertensive patients. 

In summary, five studies have demonstrated that post-operative CS values in glaucomatous eyes remained comparable to healthy subjects after MFIOL and EDOF implantation. However, one study [43] observed that glaucoma patients implanted with MFIOLs had poorer monocular visual acuity and lower CS at high spatial frequencies compared to healthy controls and patients with pre-perimetric glaucoma.

#### 3.3.3. Visual Acuity 

All 12 studies reported visual acuity outcomes. Kamath et al. [48] reported outcomes following AMO Array MFIOL (Allergan Medical Optics) implantation in 81 eyes (70 patients) with ocular pathology (study group), including 11 glaucomatous eyes and 6 eyes with ocular hypertension. The control group had implantation of monofocal IOL of similar design (AMO SI-40NB) and included 12 glaucomatous eyes. Within the study group, 29% achieved an Uncorrected Distance Visual Acuity (UDVA) of ≥6/12, while 82% achieved ≥N8 near vision, and 24% achieved ≥both 6/12 and N8 vision. Within the study group, 94% achieved a Best Corrected Visual Acuity (BCVA) of ≥6/12, while 88% achieved a BCVA of ≥N8 for near vision, and 88% achieved a BCVA of ≥both 6/12 and N8 near vision. Takai et al. [44] compared the post-operative refractive status in 20 eyes (20 patients) implanted with toric (10 eyes) and non-toric (10 eyes) IOLs during combined cataract surgery and micro-hook ab interno trabeculectomy. This study showed that the mean Uncorrected Visual Acuity (UCVA) of the Toric IOL group (logMAR 0.23 ± 0.25) was significantly better than that of the non-toric IOL group (logMAR 0.45 ± 0.26) at 3 months post-operatively (*p* < 0.05). Bissen-Miyajima et al. [41] showed that the post-operative visual outcomes (distance-corrected visual acuity, contrast sensitivity) of glaucoma patients following EDOF IOL implantation was almost comparable to those of normal eyes with the same IOLs implanted and were within normal ranges. Ichioka et al. (2021) [46] investigated the effect of toric IOL implantation on visual acuity and astigmatism in 20 POAG eyes with a pre-existing corneal astigmatism of −1.5 D, following combined cataract surgery with micro-hook ab interno trabeculotomy. Post-operatively, the logMAR UCVA was significantly better in the toric group (toric, 0.07 ± 0.07; non-toric, 0.33 ± 0.30; *p* = 0.0020). Ichioka et al. (2022) [42] investigated outcomes of toric IOL implantation on visual acuity and astigmatism in 18 POAG eyes, with a pre-existing corneal astigmatism of −1.5 D, following combined iStent implantation and cataract surgery. Pre-operatively, both groups had similar logarithm of the minimum angle of resolution (logMAR) UCVAs; post-operatively, the logMAR UCVA was significantly better in the toric group (non-toric, 0.45 ± 0.31; toric, 0.14 ± 0.15; *p* = 0.021). Ferguson et al. [40] implanted 52 POAG eyes with an EDOF IOL—(AcrySof IQ Vivity; Alcon Laboratories, Fort Worth, TX, USA) and showed favorable UDVA and Uncorrected Intermediate Visual Acuity (UIVA) at 4 months post-operatively. The mean binocular UDVA and CDVA were 0.03 ± 0.12 LogMAR and −0.06 ± 0.07 LogMAR, respectively. The mean UIVA and UNVA were 0.18 ± 0.12 LogMAR and 0.31 ± 0.18 LogMAR, respectively. A total of 85% of the subjects achieved ≥20/25 UDVA, and 77% of the subjects achieved ≥20/32 UIVA at 4 months post-operatively. Lopez-Caballero [45] compared 26 eyes undergoing iStent implantation and phacoemulsification with implantation with the AcrySof toric IOL (Alcon Laboratories, Fort Worth, TX, USA) in the study group and 41 eyes undergoing isolated phacoemulsification with toric IOL implantation in the control group. Toric IOLs were also implanted in patients with advanced visual field damage (control group: 13 mild glaucoma, 17 moderate glaucoma, 11 severe glaucoma; study group: 11 mild glaucoma, 7 moderate glaucoma, 8 severe glaucoma). There were 39 POAG, 1 closed angle glaucoma, and 2 pseudo-exfoliation glaucoma patients in the control group, while 18 POAG, 3 close angle glaucoma, 2 pseudo-exfoliation glaucoma, and 3 pigmentary glaucoma patients were included in the study group. Despite severe visual field loss, patients still achieved excellent uncorrected post-operative vision in eyes targeted for emmetropia (0.04 LogMar in the cataract group and 0.03 in the combined surgery group). Brown et al. [47] implanted AcrySof toric IOLs (Alcon Laboratories, Fort Worth, TX, USA) in 126 eyes of 87 patients with glaucoma and corneal astigmatism. The UDVA was 0.04 ± 0.08 logMAR for all eyes, and 98% of all eyes achieved an UDVA of Snellen’s 20/40 or better, with 76% achieving 20/25 or better and 47% achieving 20/20. The CDVA for all eyes was 0.01 ± 0.03 logMAR post-operatively. Rementeria-Capelo et al. [49] reported excellent visual acuity results in all patients in both the control and study groups after EDOF implantation, with both groups achieving a mean binocular uncorrected visual acuity better than 0.0 logMAR. Statistically significant differences were only found for uncorrected monocular acuity and at the +2.5 D value of the defocus curve, although these differences were unlikely to be clinically relevant. Monocular UDVA was better in the control group (−0.01  ±  0.07) compared with the study group (0.03  ±  0.08), *p* = 0.027. There were no other statistically significant differences in DVA, with an uncorrected binocular acuity of −0.06  ±  0.06 for the control group and −0.05  ±  0.06 for the study group. Kerr et al. [50] implanted EDOF IOLs (AcrySof IQ Vivity; Alcon Laboratories, Fort Worth, TX, USA) in 32 glaucomatous eyes and monofocal IOLs (Clareon/SN6ATx/SN60WF; Alcon) in 26 glaucomatous eyes. UIVA (0.06 ± 0.16 versus 0.39 ± 0.10 LogMAR; *p* < 0.001) and UNVA outcomes (0.29 ± 0.10 versus 0.55 ± 0.18 LogMAR; *p* < 0.001) were significantly better in the EDOF group than in the monofocal group, respectively. 

In summary, all studies showed excellent visual acuity results. In studies [40,44,46] comparing toric and non-toric IOLs, glaucomatous eyes implanted with toric IOLs showed better UCVA results. Studies on EDOF IOLs generally showed favorable UDVA and UIVA outcomes. However, one study [49] showed that UDVA was still better in the control group with normal eyes compared to the study group. Another study [43], examining bifocal and trifocal IOLs in 38 patients (9 glaucoma, 9 pre-perimetric glaucoma, 11 healthy), also showed that healthy patients had statistically better monocular UDVA, CDVA, and LCVA than patients with glaucoma for all values, except for binocular 10% contrast-corrected VA. Excellent uncorrected post-operative visual results were achieved even when toric IOLs were implanted in patients with advanced visual field damage. 

#### 3.3.4. Astigmatism 

Five studies [42,44,45,46,47] reported astigmatism outcomes. Ichioka et al. 2021) [46] included 20 POAG eyes (20 patients) with pre-existing corneal astigmatism exceeding −1.5 D implanted with either non-toric (n = 10) or toric IOLs (n = 10). Post-operatively, residual astigmatism was significantly less in the toric IOL group compared to the non-toric IOL group (toric, −0.63 ± 0.56 D vs non-toric, −1.53 ± 0.74 D, *p* = 0.0110; toric, 70% of eyes vs non-toric, 10% of eyes had 1.0 D or less astigmatism). Vector analyses showed the post-operative centroid magnitude of astigmatism was less in the toric IOL group (0.23 D at 83 degrees) than the non-toric IOL group (1.03 D at 178 degrees). Takai et al. [44] showed that the mean absolute residual cylinder in the non-toric IOL group (2.25 ± 0.62 D) was significantly greater than that of the toric IOL group (1.30 ± 0.68 D) (*p* < 0.05). Post-operatively, 60% of eyes in the toric IOL group and 10% in the non-toric IOL group had an absolute astigmatism level of 1.5 D or less. Brown et al. [47] showed that toric IOLs can reliably reduce astigmatism and improve uncorrected vision in eyes with cataract and glaucoma. Astigmatism improved from 1.47 ± 1.10 D to 0.31 ± 0.37 D post-operatively. The residual cylinder was 1.00 D or less in 97% of eyes, 0.75 D or less in 90% of eyes, and 0.50 D or less in 83% of eyes. Ichioka et al. (2022) [42] included 18 POAG eyes with pre-existing corneal astigmatism exceeding −1.5 D implanted with non-toric (n = 10) or toric (n = 10) IOLs. Astigmatism decreased significantly in the toric group post-operatively compared to the non-toric group (non-toric, −2.03 ± 0.63 D; toric, −0.67 ± 0.53 D; *p* = 0.0014) Vector analyses showed the post-operative centroid magnitude and confidence eclipses of astigmatism was less in the toric group (0.47 D at 173° ± 0.73 D) than the non-toric group (1.10 D at 2° ± 1.91 D). Post-operatively, 78% of eyes in the toric group had 1.0 D or less refractive astigmatism compared with 11% in the non-toric group. Lopez-Caballero [45] compared 26 eyes with iStent and toric IOL implantation in the study group and 41 eyes undergoing isolated phacoemulsification with toric IOL implantation in the control group. The mean post-operative refractive cylinder was 0.26 D in the control and 0.11 D in the iStent group.

In summary, studies have demonstrated that toric IOLs provide predictable and good astigmatism outcomes in glaucomatous eyes undergoing standalone cataract surgery or in combination with selected glaucoma surgeries. 

#### 3.3.5. Patient Satisfaction

Five studies [22,40,43,49,50] reported on patient satisfaction via visual questionnaires. Ferguson et al. [40] implanted 52 eyes (26 patients) with POAG, with EDOF non-toric or toric IOLs (AcrySof IQ Vivity or AcrySof IQ Vivity Toric (Alcon Laboratories, Fort Worth, TX, USA)) and found that 85% of subjects reported they would choose the same lens again. Sanchez-Sanchez et al. [43] concluded that MFIOLs may be implanted in patients with pre-perimetric glaucoma with little fear of patient dissatisfaction. Interestingly, Rementeria-Capelo et al. [49] reported that all patients in the study group (including glaucoma patients) had a higher satisfaction with their visual performance than patients in the control group (average satisfaction in control group: 3.52  ±  0.51; study group: 0.84  ±  0.37, 52% (*p* = 0.016), and patients in the control group reported greater difficulty in reading newspapers (*p* = 0.030). All patients stated they would undergo surgery again with the same type of IOL. Kerr et al. [50] implanted EDOF IOLs (AcrySof IQ Vivity; Alcon Laboratories, Fort Worth, TX, USA) in 32 glaucomatous eyes and monofocal IOLs (Clareon/SN6Atx/SN60WF; Alcon) in 26 glaucomatous eyes. Patient satisfaction was significantly higher in the EDOF group for distance, intermediate, and near vision than in the monofocal group. All EDOF patients were “very satisfied” with their distance and intermediate vision compared to 9/13 (69.2%) and 6/13 (46.2%) in the monofocal group, respectively. For near vision, 12/16 (75.0%) in the EDOF group were “very satisfied” with their unaided near vision compared to 5/17 (38.5%) in the monofocal group (*p* = 0.059). Patients who received an EDOF lens were more likely to report that they would choose the same lens again (16/16 (100%) in the EDOF group compared to 10/13 (76.9%) in the monofocal group; *p* = 0.085). 

Ouchi et al. [22] evaluated outcomes of 11 patients (15 eyes with coexisting ocular pathologies, 1 eye with past history of acute angle closure of Aulhorn classification stage 3 glaucoma, and 3 NTG eyes of Aulhorn classification stage 3) that underwent implantation of LENTIS Mplus (Oculentis GmbH). No patient reported poor or very poor vision quality. For distance vision, all 11 patients (100%) rated their quality of vision as 4 or higher (very good or good) among 5 items. For near vision, all 11 patients (100%) rated their quality of vision as 3 (acceptable) or higher.

In summary, studies have suggested a high level of patient satisfaction following the implantation of various premium IOLs in glaucoma patients, with two studies [49,50] reporting higher post-operative satisfaction in glaucomatous eyes compared to control eyes and a high percentage of patients stating they would choose the same IOL again.

#### 3.3.6. Glares and Haloes

Three studies reported on glare outcomes. Ferguson et al. [40] implanted 52 POAG eyes with EDOF non-toric or toric IOLs and assessed glares and haloes reported on a scale of 1–5 (1 = not at all, 5 = extremely). Subjects reported a mean response of 2.6 ± 1.3 when asked if they noted glare/halos in dim light situations. However, 65% of the subjects reported not being bothered or only having very little dissatisfaction with glare/halo symptoms. Bissen-Miyajima et al. [41] evaluated outcomes of diffractive EDOF IOLs in 16 NTG eyes. Most cases reported the severity of glares, halos, and starbursts as none, mild, or moderate. Only one subject reported severe halos and another reported severe starburst. Kerr et al. [50] implanted EDOF IOLs in 32 glaucomatous eyes and monofocal IOLs in 26 glaucomatous eyes. Most participants did not experience any photic phenomena (glares/halos/starbursts), and there was no significant difference in the incidence of photic phenomena between both groups. Seven patients in the EDOF group reported glare, compared to six patients in the monofocal group. 

Overall, across the three studies examining EDOF IOL implantation, despite subjects often reporting at least some level of glare or halo effects, many were not significantly bothered by these symptoms. There was no significant difference in photic phenomena between EDOF and monofocal IOLs in glaucomatous eyes. However, in one study, ocular pathology appeared to be associated with an increased incidence of halos.

## 4. Discussion: Considerations in Premium IOL Implantation in Glaucoma 

Published literature has discussed a range of factors that may be considered when contemplating premium IOL implantation in glaucoma eyes.

### 4.1. Contrast Sensitivity 

Contrast sensitivity (CS) is the measure of an individual’s ability to detect a difference in luminance between two areas [51] and, in this context, may be affected by various factors including both the IOL and glaucoma. Decreased CS in glaucoma patients have been well documented [51,52,53]. Already in early disease, patients begin to lose retinal ganglion cells and retinal nerve fibre layer (RNFL) thickness, resulting in structural and functional changes causing a decrease in contrast sensitivity (CS). Glaucoma preferentially affects CS more than visual acuity (VA), and the decrease in CS correlates with the degree of structural and functional glaucomatous damage [51]. CS losses occur even in individuals with minimal or no field loss (<3 dB) and a relatively good VA (0.3 log MAR or better) [53]. CS has been found to correlate with visual field (VF) sensitivity and affects vision-related quality of life [53,54]. At mesopic levels, CS is correlated with visual field loss and affects glaucoma patients ability to perform daily activities and negatively impacts their quality of life [55]. 

MFIOLs, particularly refractive MFIOLs [6], have been shown to result in decrease in CS, where the mesopic CS is worse than photopic sensitivity, and where the loss is greater at higher compared to lower spatial frequencies after MFIOL implantation [56].

Farid et al. [19] stated that as the amount of light energy in focus at any given focal distance is reduced, out-of-focus light is superimposed, and approximately 18% of transmitted light in diffractive IOLs (which may vary depending on IOL design) is lost to higher orders of diffraction that are never focused on the retina. As a result, patients with multifocal IOLs may experience glare and halos, as well as reduced contrast sensitivity.

Hence, while MFIOLs may be considered in patients with early glaucoma or controlled ocular hypertension, they should be avoided in patients with uncontrolled and advanced glaucoma [18]. Cao et al. [20] found that both refractive and diffractive MFIOL subgroups had a lower CS, and Hawkins et al. [51] showed that reduced CS is significantly correlated with visual field losses in patients with glaucoma. 

Nonetheless, studies implanting MFIOLs in glaucomatous eyes have shown promising results. Sanchez Sanchez et al. [43] found no clinically significant difference in CS between eyes with pre-perimetric glaucoma and healthy controls after MFIOL implantation. Ouchi et al. [22] showed that following MFIOL implantation, CS in all eyes with ocular pathologies were comparable to that of healthy eyes, and concluded that with careful case selection, sectorial refractive MFIOL may be effective in eyes with concurrent ocular pathology. Furthermore, ongoing developments in IOL technology such as IOL asphericity, used both in monofocal IOLs and premium IOLs today, have also improved CS outcomes after cataract surgery. Trueb et al. [57] demonstrated that eyes implanted with the aspheric AcrySof IQ IOL had better photopic and mesopic CS at medium and high spatial frequencies than in eyes implanted with the spherical AcrySof SN60AT IOL. Deshpande et al. [58] demonstrated that the optical design of aspheric IOLs reduced spherical aberrations and increased CS. Alternatively, accommodative IOLs may be considered advantageous as these lenses do not depend on pupil size and do not decrease CS [34]. However, they have increased risk of capsular contraction, commonly seen in pseudo-exfoliation patients, a condition associated with weakened zonules, further decreasing the functionality of IOL accommodative system [34]. 

### 4.2. Glaucomatous Visual Field (VF) Defects

The severity, extent, and location of glaucomatous VF defects are also considered when deciding if a glaucoma patient would benefit from premium IOLs. 

Studies have utilized Octopus 101 autoperimetry, Goldmann manual perimetry, frequency doubling technology matrix perimetry, the automated Esterman binocular field test, and Humphrey Visual Field 30-2 perimetry testing in exploring visual field outcomes after MFIOL implantation.

Prior reviews have suggested that only glaucoma suspects and ocular hypertensive patients with no optic disc or visual field damage who have been stable for a longer period of time should be candidates for MFIOL implantation [6,34]. In addition, good control and stability of visual field damage, with no evidence of progression, has also been suggested to be a necessary prerequisite for premium IOL implantation in glaucomatous eyes [34]. With respect to lens choice, two recent studies [19,59] have shown that MFIOLs decrease the mean deviation (MD) of visual field tests with the Humphrey field analyzer (Carl Zeiss Meditec, Jena, Germany) compared to diffractive bifocal IOLs, monofocal IOLs or phakic eyes.

Farid et al. [19] demonstrated a significant depression of approximately 2 dB in HVF 10-2 testing in healthy eyes which underwent diffractive MFIOL implantation compared to monofocal IOL implantation. Aychoua et al. [59] demonstrated similar results on HVF 30-2 testing and concluded that it was likely due to reduction in differential light sensitivity [60]. Kang et al. [61] supported this finding, showing that patients with MFIOLs (3M diffractive bifocal IOL) have greater reduction of visual field on the Goldmann manual perimetry, compared to patients with monofocal IOLs, and this was reflected across different spot sizes and intensities. 

However, Bi et al. [62] examined differences in Octopus 101 autoperimetry results between patients with MFIOLs (AcrySof ReSTOR SA60D3) and patients with monofocal IOLs (AcrySof SN60AT) and found no significant difference between the two groups. 

### 4.3. Glaucoma Subtype

The varying characteristics of different subtypes of glaucoma may also influence the decision of premium IOL implantation in eyes with glaucoma. Within the spectrum of angle-closure disease, primary angle closure suspects (PACS) have no glaucomatous nerve damage nor functional visual defects, may experience angle opening after cataract extraction, and may benefit from premium IOLs. It should be noted however, that prior laser peripheral iridotomy is associated with a higher risk of zonulysis [63] and lens subluxation [64,65], which would pose challenges to premium IOL implantation. Eyes with established primary angle closure glaucoma (PACG) have functional VF defects and are also at a higher risk of disease progression compared to eyes with POAG [66,67], and this should be taken into consideration, even if there is only mild disease severity at baseline. In addition, angle-closure disease is associated with shorter axial length. This may increase the risk of refractive surprise and may be a consideration when choosing premium IOLs, although this may be much less of a concern today given the significant improvements in biometry and lens calculation methods.

Pseudo-exfoliation glaucoma (PXG) is a form of secondary open angle glaucoma that arises due to the deposition of extracellular material in the anterior chamber, trabecular meshwork, and other tissues in the eye [34], resulting in raised IOP and glaucomatous optic nerve damage. These eyes have poorly dilating pupils, a higher risk of zonular instability, a higher risk of uncontrolled IOP post-operatively [68], and may experience greater post-operative inflammation from vascular instability [69]. These factors are likely to pose challenges to premium IOL implantation and diminish their benefit with suboptimal post-operative refractive outcomes. First, intra-operatively, poorly dilating pupils limit the view of axis markings on toric IOLs and require additional pupil maneuvering during surgery. Second, zonular dialysis, posterior capsule rupture [70], and vitreous loss [71,72] which may occur during surgery often precludes the implantation of premium IOLs in these eyes. Third, the increased risk of posterior capsular opacification and capsular phimosis over time, with progressively weakening zonular support, has been shown to lead to a higher rate of IOL subluxation [73]. PXG is associated with significant IOL axis misalignment [74] and has been identified as the most common cause of IOL dislocation, often occurring less than a decade even after uncomplicated cataract surgery [75,76]. Late in-the-bag spontaneous intraocular lens dislocation [77] and progressive IOL decentration are not uncommon [78]. Hence, premium IOLs, which require precise centration and whose refractive outcomes are particularly affected by anterior capsule phimosis and zonulysis, have been mostly avoided in PXG eyes [34,79]. PXG eyes have an increased risk of refractive surprise after phacoemulsification: Manoharan et al. [80] analyzed refractive outcomes of phacoemulsification cataract surgery in glaucoma patients and showed that the odds of refractive surprise being greater than ±1.0 D were higher in patients with pseudo-exfoliation glaucoma (n = 23 eyes) compared with patients without glaucoma (OR = 7.27, *p* = 0.0138). Fourth, PXG has a greater risk of progression compared to other glaucoma subtypes following cataract surgery, the result of uncontrolled IOP and greater IOP fluctuation post-operatively [81]. Hence, implantation of premium IOLs even in PXG eyes with mild glaucoma should be cautioned against. Finally, the higher rate and greater severity of post-operative inflammation, iritis, and cellular precipitates [71] in these eyes may compromise visual acuity and reduce the benefit of premium IOL implantation in eyes with PXG.

### 4.4. Ocular Surface Disease (OSD)

OSD has been found to be present in 59% of glaucoma patients [82], with its incidence related to previous or ongoing conjunctival inflammation and scarring, tear film instability, advanced age, and the use of chronic anti-glaucoma medications. Glaucoma itself is associated with dry eye disease (DED), with approximately 11% of 5 million Americans over 50 years old found to have coexisting glaucoma and DED [83]. Untreated POAG patients also have a higher risk of ocular surface disease due to a 22% lower basal tear turnover rate, compared to patients without glaucoma [84]. The severity of OSD appears to increase with worsening glaucoma severity and results in decreasing quality of life [85]. Chronic anti-glaucoma medications themselves are a risk factor for OSD [83]. Prostaglandin analogues, for example, have been associated with meibomian gland dystrophy (MGD) [86], which leads to unstable tear film and fluctuating vision. 

Severe OSD and DED are associated with inaccurate biometry and topography, which may result in inaccurate IOL selection [87,88]. Hence, the ocular surface would need to be carefully evaluated and optimized prior to biometry to ensure accurate ocular measurements and IOL power calculation, which is crucial in premium IOL selection. Post-operatively, OSD would still require continual management to ensure maximal visual benefit from premium IOL implantation. Fortunately, the IOP-lowering effect of cataract surgery itself may reduce the glaucoma medication burden post-operatively, thereby alleviating OSD and improving patients’ quality of life after surgery [89].

### 4.5. Axial Length (AL) and Anterior Chamber Depth (ACD) 

Successful pseudo-phakic rehabilitation after combined cataract and glaucoma surgery requires accurate IOL power calculation, which depends on precise measurements of the AL and ACD [90]. Eyes with glaucoma are associated with extremes in AL, with open angle glaucoma associated with high myopia [91] and increased AL [92], while angle-closure eyes have been shown to have shorter ALs [93]. Extreme ALs may, in the past, have been associated with increased risk of IOL calculation errors; however, modern IOL formulae have proven accurate even for extreme axial lengths, and they may lessen the influence of AL on the accuracy of IOL power calculations [94,95]. In a network meta-analysis, Lu et al. [95] investigated eight formulae: including Barrett Universal II, Kane, SRK/T, Hoffer Q, Haigas, Holladay I, Hill-Radial Basis Function (RBF 3.0) and Ladas Super Formula (LSF) used for IOL power calculations in eyes with PACG and found no significant differences in outcomes among all the above formulae. Studies have also shown that greater changes in ACD may occur post-operatively in eyes with a pre-operatively shallow ACD (<2.5 mm) and a shorter AL (<23 mm), and that this was associated with a higher risk of refractive error after cataract surgery [96]. 

### 4.6. Type of Glaucoma Surgery 

#### 4.6.1. Combined Phacoemulsification and Trabeculectomy Surgery

Combined phacoemulsification and trabeculectomy surgery (“phacotrabeculectomy”) improves visual acuity and minimizes the post-operative IOP spikes and morbidity that may occur in a two-stage operation [97,98]. Previous studies have shown that phacotrabeculectomy can provide an IOP reduction that is not inferior to that of trabeculectomy alone [99,100], with visual outcomes following this procedure being comparable to those obtained with phacoemulsification alone [101,102]. However, they have a higher risk of complications such as hypotony, hyphema, and anterior chamber shallowing compared to phacoemulsification alone [103], and they are also associated with surgically induced astigmatism which may affect refractive outcomes [104]. There are also various aspects of phacotrabeculectomy surgery that may affect the post-operative ocular characteristics and refractive status of the eye, thus increasing the risk of refractive surprises that may therefore preclude the implantation of premium IOLs in glaucoma eyes.

First, multiple studies [105,106,107] have suggested a greater risk of myopic shift and myopic refractive surprise following phacotrabeculectomy compared to phacoemulsification alone. Chan et al. [107] demonstrated that phacotrabeculectomy was more likely to have an IOL prediction error of >1.00 D (*p* = 0.02) and a myopic shift of >0.50 D or 1.00 D (*p* = 0.03 or 0.02, respectively) post-operatively. This observed myopic shift may be attributed to the trabeculectomy surgery itself [108,109,110]. While Muallem et al. [108] and Zhang et al. [110] assessed the performance of ocular biometry via contact A-scan ultrasonography, and Tan et al. [109] and Zhang et al. [110] performed calculations using early generation IOL, Yeh et al. [111], using the Haigis formula, also found a higher frequency of myopic surprise (>1.0 D) in post-trabeculectomy eyes with pre-operative IOP <9 mm Hg despite the use of laser interferometry as well as modern biometry and IOL prediction formulae. Additionally, post-operative myopic surprises appeared to be associated with IOP spikes—37.5% of eyes with pre-operatively low IOPs and subsequent IOP spikes experienced myopic surprises of over 1 D. These refractive changes after trabeculectomy surgery are likely to negatively affect the accuracy and predictability of visual outcomes following premium lens implantation in phacotrabeculectomy surgery.

Second, despite its overall good safety profile, phacotrabeculectomy has its associated complications, including hypotony and hypotonous maculopathy, hyphema, anterior chamber shallowing, and visual field wipeout. The frequency of these complications are higher than that of phacoemulsification alone [103,112,113], with many potentially causing refractive errors or even persistent visual loss after surgery [114,115]. 

Third, changes in AL and ACD [116,117,118] have been reported to occur after trabeculectomy surgery, thus increasing the risk of IOL power calculation errors [107,119] despite accurate pre-operative ocular biometry measurements [120]. A decrease in ACD may cause the eye to be more myopic post-operatively, while a decrease in AL would render the eye more hyperopic [106]. Studies have demonstrated a decrease in AL after phacotrabeculectomy; Poon et al. [121] showed that phacotrabeculectomy resulted in a mean decrease in AL of 0.16 ± 0.15 mm in PACG and 0.16 ± 0.11 mm in POAG eyes (*p* = 0.96), as well as an increase in ACD of 1.41 ± 0.91 mm in PACG, and 0.87 ± 0.86 mm in POAG eyes (*p* = 0.04). Law et al. [122] demonstrated that AL reduction following phacotrabeculectomy (117 (57) microm) was significantly larger than that following cataract surgery alone (75 (38) microm, *p* < 0.02), and this correlated significantly with post-operative IOP (*p* < 0.002). This decrease in AL is likely due to the effect of the trabeculectomy surgery itself, as demonstrated in a number of previous studies [116,117,123]. Factors that may affect AL include post-operative IOP changes and methods used to measure AL. Studies [116,123,124] have shown that the magnitude of AL change may depend on the magnitude of IOP change. Studies that use applanation ultrasound have reported larger reductions in AL after trabeculectomy [116,117,125], and different methods used to measure AL may explain disparity between studies, with any difference possibly being due to avoidance of globe indentation with the non-contact method, in contrast to contact ultrasonic biometry that may underestimate AL in soft, post-trabeculectomy eyes [116,117,118,123,126]. Francis et al. [127] utilized non-contact optical biometry and found a small but statistically significant decrease in AL following trabeculectomy, but with a difference less than that seen in the above-mentioned studies [116]. Of note, however, Zhang et al. [110] obtained AL measurements of patients undergoing phacoemulsification after prior trabeculectomy using both non-contact and contact methods of AL measurement and found no significant difference in either mean AL or mean ACD measured by both methods. 

Lastly, keratometry readings may be affected by trabeculectomy surgery [125,128,129]. Various studies have demonstrated astigmatism, superior steepening, or superior flattening, with effects lasting up to 12 months post-operatively [104,117,122,129,130,131,132,133,134,135]. Trabeculectomies performed in the superior quadrant may induce the axis of corneal astigmatism to fall at the meridian of the scleral flap, causing a with-the-rule (WTR) corneal astigmatism [104,130,136]. Hong et al. [137] reported a shift of astigmatism at the vertical meridian from +2.17 D to a −1.72 D over 12 months after a combined triple procedure of a trabeculectomy, extracapsular cataract extraction, and IOL implantation. A study that evaluated keratometric changes after trabeculectomy revealed induced WTR astigmatism with a mean of 0.81 ± 1.08 D at the post-operative month three which tends to resolve within one year [134]. 

In comparing post-trabeculectomy patients undergoing standalone cataract surgery and patients undergoing phacotrabeculectomy, Bae et al. [106] found that IOL power prediction was less accurate in OAG patients undergoing cataract surgery post-trabeculectomy (CAT group) or undergoing combined phacotrabeculectomy surgery (CCT group) compared to OAG patients undergoing standalone cataract surgery (OC group). A prior trabeculectomy resulted in larger refractive prediction error, while combined trabeculectomy resulted in a slight myopic shift post-operatively. In the CAT group, the large mean absolute error (MAE) could have been due to contact A-scan ultrasonography used to measure AL as these softer, post-trabeculectomy eyes were more susceptible to deformation, thereby resulting in a falsely shorter AL measurement and greater myopic refractive surprise [111]. In the CCT group, the mean error was approximately −0.40 D, which was statistically greater than that in the OC group. Phacotrabeculectomy causes a greater IOP change compared to staged cataract surgery following prior trabeculectomy (6.61 versus 0.59 mmHg) [128], thus potentially causing greater AL, ACD, and keratometry changes [113,124]. 

In summary, biometric changes may occur after trabeculectomy surgery and should be a consideration in patients undergoing phacotrabeculectomy or standalone cataract surgery after prior trabeculectomy. AL, ACD, and keratometry are primary determinants in IOL power calculations; hence, post-operative changes in these biometric measurements may lead to increased risk of post-operative refractive surprise and IOL power calculation errors [127,138], although some studies have demonstrated that post-phacotrabeculectomy AL and keratometric changes may effectively negate each other’s effect [139]. Standalone cataract surgeries performed much later (at least 3–6 months [118]) after initial trabeculectomy surgery may allow refractive outcomes and ocular measurements to stabilize, thereby not precluding the use of premium IOLs (particularly toric IOLs) in these patients. The use of non-contact optical biometry [38], such as laser interferometry and modern biometry formulae [140], may provide more accurate IOL power calculation for eyes undergoing combined phacotrabeculectomy.

#### 4.6.2. MIGS (Minimally Invasive Glaucoma Surgery)

MIGS are a newer spectrum of surgical techniques and devices which have emerged rapidly in recent years. Most share common characteristics, including having a micro-invasive approach, minimal tissue trauma, at least modest efficacy, as well as a more rapid post-operative recovery and higher safety profile compared to traditional glaucoma surgeries [141]. MIGS can be classified based on the site of anatomical intervention and augmentation, including (1) angle-based MIGS, where trabecular outflow is increased by bypassing the trabecular meshwork and directing aqueous humor into Schlemm’s canal; (2) subconjunctival MIGS, where a drainage pathway is created into the sub-Tenon’s space; and (3) suprachoroidal MIGS, where uveoscleral outflow is increased via implantation of suprachoroidal shunts [142]. Premium IOL implantation, as explained in the aforementioned sections, requires refractive neutrality and ocular biometric stability of surgical procedures. A number of studies have examined refractive outcomes following cataract surgery and monofocal IOL implantation with MIGS. However, few have reported results of premium IOL implantation in this context.

Angle-based MIGS bypass the resistance to aqueous outflow at the level of the trabecular meshwork, through microstenting, micro-incisions, and viscodilation [143]. Microstenting options include the iStent series. The first-generation iStent (Glaukos Corp., San Clemente, CA, USA) was introduced in 2012, with clinical trials demonstrating its efficacy when implanted in combination with cataract extraction. Samuelson et al. [144] showed significant IOP reduction after iStent and cataract surgery compared to cataract surgery alone, with similar safety profiles. Scott et al. [145] demonstrated that combined iStent and cataract surgery is likely to be a refractively neutral procedure—80% and 95% of eyes achieved target refraction within ±0.5 D and ±1.00 D, respectively. Manoharan et al. [80] demonstrated no difference in refractive outcomes in glaucoma patients who underwent combined phacoemulsification with iStent compared to phacoemulsification alone. The later iteration of the iStent, the iStent Inject (Glaukos Corp., San Clemente, CA, USA), achieved US FDA approval in 2018. Ang et al. [146] demonstrated minimal influence of iStent inject implantation on the MAE (−0.13 ± 0.08 D), with 82.8% of eyes achieving a post-operative refraction within 0.5 D of target in combined iStent inject implantation and phacoemulsification in Asian eyes with NTG. Ioannidis et al. [147] concluded too that the iStent inject device is refractively neutral—73.9% and 98.9% of eyes were within 0.5 D and 1.0 D of the target refraction, respectively. Trabecular bypass stents would not be expected to impact refractive outcomes, given that multiple studies [40,42,45,144] have demonstrated the refractive neutrality of this device. Other angle-based MIGS include micro-incision options, such as the Trabectome and Kahook Dual Blade. The Trabectome (NeoMedix, Inc., Tustin, CA, USA) was FDA approved in April 2004 [148]. Luebke et al. [149] demonstrated no difference in refractive outcomes between patients undergoing combined trabectome–cataract surgery compared to cataract surgery alone. Refractive outcomes following Kahook Dual Blade (New World Medical, Rancho Cucamonga, CA, USA) surgery, a goniotomy procedure, was also studied by Sieck et al. [150], who demonstrated no difference in refractive outcomes of glaucomatous patients undergoing phacoemulsification with or without KDB goniotomy. 

Subconjunctival MIGS options include the XEN45 Gel Stent (Allergan, Dublin, CA, USA) and Preserflo Microshunt (Santen Co., Japan). The XEN45 Gel Stent was FDA approved in 2016 [148], and Grover et al. [151] demonstrated a significant reduction in IOP and medication use with a good safety profile, thus offering XEN45 as a MIGS option for patients with refractory open-angle glaucoma. Bormann et al. [152] compared refractive changes after surgery between trabeculectomy and the XEN45, showing that the SIA was nearly similar in both groups (0.75 ± 0.60 diopters after TE and 0.81 ± 0.56 diopters after XEN; *p* = 0.57).

Suprachoroidal MIGS procedures aim to take advantage of the uveoscleral pathway to reduce IOP, not being subject to an episcleral venous pressure floor [142]. As they have the most potential to alter the AL of the eye, they may result in post-operative refractive surprises. Although there are no US FDA-approved suprachoroidal MIGS at present, there are multiple devices in the investigational pipeline, such as the iStent Supra (Glaukos Corporation, San Clemente, CA, USA) and the MINIject (iStar Medical, Wavre, Belgium) [142]. Previously, the Cypass (Alcon, Ft. Worth, TX, USA) showed significant IOP and medication reduction when combined with cataract extraction [153] but was withdrawn from the market in 2018 when 5-year data from the COMPASS-XT study suggested a significantly increased rate of endothelial cell loss [154]. While there are no formal published studies to support these claims, there have been numerous independent accounts on various platforms reporting myopic surprises after combined Cypass and cataract extraction, with anecdotal accounts reporting myopic shifts of between 1.00 D to 3.00 D [155]. 

As largely refractively neutral surgeries with a high safety profile, angle-based MIGS appears less subject to confounding factors that may influence post-operative refractive outcomes; hence, it may potentially be favorable to the implantation of premium IOLs. Toric IOL implantation appears suitable in angle-based MIGS, with studies demonstrating good refractive results and spectacle-free outcomes following cataract extraction and toric IOL implantation with Tanito microhook trabeculotomy (TMH) [44,46] and iStent implantation [45,46,47]. Subconjunctival MIGS, performed ab internally without requiring conjunctival closure, is less likely to induce significant SIA, resulting in more predictable and consistent refractive outcomes, which contrasts with trabeculectomy which increases WTR astigmatism post-operatively [127]. 

### 4.7. Functional and Structural Monitoring of Glaucoma

MFIOLs may affect the sensitivity of investigative tests for glaucomasu progression, including visual field assessment and optic nerve imaging. Inoue et al. [156] reported that diffractive MFIOLs may cause wavy artifacts on OCT imaging. Aychoua et al. [59] reported that patients with diffractive MFIOLs (Tecnis^®^ Multifocal ZM900; AMO, AT LISA^®^ 809M; CarlZeiss Meditec, Jena, Germany) demonstrated a clinically relevant reduction in visual sensitivity (with a lower mean deviation of 2 dB, compared to monofocal IOLs) as assessed with standard automated perimetry, and the study concluded that the reduction was likely related to the MFIOL design, as opposed to patients’ pseudo-phakic status. Furthermore, in patients with posterior eye segment changes, the visualization of macula and the optic nerve may be impaired after both toric and MFIOL implantation, and this could pose difficulties in later diagnostic or therapeutic procedures [157].

## 5. Conclusions

Overall, few studies have explored the use of premium IOLs in glaucoma patients, with some studies including only a small number of glaucomatous eyes. However, results thus far are promising. Studies have demonstrated high spectacle independence (especially for distance vision), contrast sensitivity comparable to that of healthy subjects, and excellent visual acuity results. Toric IOLs have been shown to provide good visual and refractive outcomes in eyes undergoing selected glaucoma surgeries and in eyes with ocular hypertension and incipient glaucoma, with high levels of patient satisfaction post-operatively. However, there may currently be insufficient evidence to support the safety of premium IOL implantation in patients with advanced glaucoma. Caution should be applied when cataract extraction is combined with certain glaucoma surgeries as there may be additional intra- and post-operative factors that influence the quality of vision after surgery.

Premium IOLs may confer greater benefit with appropriately chosen patient profiles [158] and may be recommended for certain patients with glaucoma, depending on the disease severity and type of visual field deficit. Specifically, premium IOLs tend to have better outcomes in glaucoma suspects, patients with ocular hypertension, and glaucoma patients with early, well-controlled disease. IOL selection should be individualized according to the patient’s desired refractive outcome, visual expectations, subtype of glaucoma, type and severity of glaucomatous visual field defect, presence of ocular surface disease, and type of glaucoma surgery. Regular reviews on this topic are necessary given the rapid advancements in both IOL technology and glaucoma surgical treatment [159].

## Figures and Tables

**Table 1:** Inclusion and exclusion criteria.

Inclusion Criteria	Exclusion Criteria
Population: Patients with visually significant cataract (unilateral or bilateral) and glaucoma. Studies: Randomized controlled trials, case series, prospective and retrospective studies. Interventional Arm: Phacoemulsification or femtosecond laser cataract surgery with premium IOL implantation, performed with or without concurrent glaucoma surgery.	Population Primary surgeries other than phacoemulsification with intraocular lens implant and glaucoma surgery (e.g., corneal inlays); Secondary surgeries; Concomitant ocular pathology besides glaucoma: keratopathy, maculopathy, retinopathy, optic neuropathy, as well as any ocular condition that is deemed to confound visual acuity assessment; Non-visually significant cataracts (e.g., clear lens). Studies Non-published studies; Studies not written in English. Interventional Arm Clear lens extraction or refractive lens exchange; Extracapsular cataract extraction; Intracapsular cataract extraction.

**Table 2:** Quality assessment using the Newcastle–Ottawa scale of included cohort studies (included studies: [3,4,5,6,7,8,9,10]).

Author (Year)	Modified Newcastle-Ottawa Scale								
	Selection				Comparability	Outcome			Total Score
	Representativeness of Exposed Cohort (Maximum:⋆)	Selection of Non-Exposed Cohort (Maximum:⋆)	Ascertainment of Exposure (Maximum:⋆)	Demonstration that the Current Outcome of Interest Was Not Present at Start of Study (Maximum:⋆)	Comparability of Cohorts on the Basis of the Design or Analysis (Maximum:⋆)	Assessment of Outcome (Maximum:⋆)	Was Follow Up Long Enough for Outcomes to Occur (Maximum:⋆)	Adequacy of Follow Up of Cohorts (Maximum:⋆)	
Ichioka 2022 [4]	⋆		⋆	⋆	⋆	⋆	⋆	⋆	7
Sanchez-Sanchez 2021 [7]	⋆	⋆	⋆	⋆	⋆	⋆	⋆	⋆	8
Takai 2021 [10]	⋆	⋆	⋆	⋆	⋆	⋆	⋆	⋆	8
Lopez Caballero 2022 [6]	⋆	⋆	⋆	⋆	⋆	⋆	⋆	⋆	8
Ichioka 2021 [3]	⋆		⋆	⋆	⋆	⋆	⋆	⋆	7
Kamath 2000 [8]	⋆	⋆	⋆	⋆	⋆	⋆	⋆	⋆	8
Rementería-Capelo 2022 [5]	⋆	⋆	⋆	⋆	⋆	⋆	⋆	⋆	8
Kerr 2023 [9]	⋆		⋆	⋆	⋆	⋆	⋆	⋆	7

**Table 3:** Quality assessment using the modified Newcastle–Ottawa scale (M-NOS) for other included studies (included studies: [11,12,13,14]).

Author (Year)	* Selection	Ascertainment	Causality					Reporting
	Q1	Q2	Q3	Q4	Q5	Q6	Q7	Q8
Bissen Miyajima 2023 [11]	No	Yes	Yes	No	No	No	Yes	Yes
Brown 2015 [12]	No	Yes	Yes	No	No	No	Yes	Yes
Ouchi 2015 [13]	No	Yes	Yes	No	No	No	Yes	Yes
Ferguson 2023 [14]	No	Yes	Yes	No	No	No	Yes	Yes

* M-NOS components.

## Supplementary Materials

The following supporting information can be downloaded at https://www.mdpi.com/article/10.3390/bioengineering10090993/s1: Table S1: Detailed Search Strategy; Table S2: Patient and Study Characteristics; Table S3: Visual Results Table; Table S4: Refractive Outcomes Table.
