# Supplementary material for: Premium Intraocular Lenses in Glaucoma—A Systematic Review
Source: Bioengineering (Basel). 2023 Aug 22;10(9):993. doi: 10.3390/bioengineering10090993 (PMC10525961; doi:10.3390/bioengineering10090993)
Supplement: Supplementary file 1 [file bioengineering-10-00993-s001.zip › Supplementary materials_Premium IOL/SUPP TABLES - Premium Intraocular Lenses in Glaucoma - A Systematic Review - Bryan290623 FINAL.docx]

**Supplementary Material**

**Table S1: Detailed Search Strategy**

| **1** | **(multifocal* or bifocal* or trifocal* or diffractive* or toric* or accomodating* or IOL or EDOF or "multifocal lens*" or multifocality or "multifocal intraocular lens*" or EDOF or "extended depth of focus*" or "Extended Depth-of-Focus").ti,ab.** |
| --- | --- |
| **2** | **exp Glaucoma/ or glaucoma.ti,ab.** |
| **3** | **1 AND 2** |

**Table S2: Patient and Study Characteristics**

Includes patient demographics and clinical characteristics as well as major study characteristics, including surgical procedures and type of lens placed.

|  |  |  | **Country** | **Number of Patients (Number of eyes)** | | **Age** | | **Gender (M/F)** | |  |  |
| --- | --- | --- | --- | --- | --- | --- | --- | --- | --- | --- | --- |
| **Author/Year** | **Pathology** | **Lens Type** |  | **Control (n)** | **Subject Patients (n)** | **Control (Mean, SD)** | **Subject Patients (Mean, SD)** | **Control** | **Subject Patients** | **Type of Surgery** | **Evaluation Time (months)** |
|  |  |  |  |  |  |  |  |  |  |  |  |
| Ferguson 2023[40] | mild, pre-perimetric OAG | EDOF: non-diffractive, wavefront-shaping EDOF non-toric or toric IOL (AcrySof IQ Vivity or AcrySof IQ Vivity Toric) | US | NA | 26 (52 eyes) | NA | 69.4 ± 6.6 | NA | 12/ 14 | 23% phaco, 77% phaco + trabecular microbypass stent | 4 |
|  |  |  |  |  |  |  |  |  |  |  |  |
| Bissen-Miyajima 2023[41] | NTG (5/16 categorized severe in the HPA) | EDOF: Symfony®, models ZXR00V and ZXV150-375 | Japan | NA | 10 (16 eyes) | NA | 66.5± 7.1 | NA | 5/5 | femtosecond laser-assisted cataract surgeries | 3 |
|  |  |  |  |  |  |  |  |  |  |  |  |
| Ichioka 2022[42] | OAG with preexisting corneal astigmatism of -1.5 diopter (D) or more | non-toric IOL (Vivinex iSert XY1, Hoya, Tokyo, Japan; n = 9 (7 POAG, 2 EXG)) or a toric IOL (Vivinex Toric XY1, Hoya; n = 9 (9 POAG)) | Japan | Non Toric IOL: 9 (9 eyes) | Toric IOL: 9 (9 eyes) | Non Toric: 82.6 ± 8.2 | Toric: 78.3 ± 6.4 | Non Toric: 6/3 | Toric: 5/4 | combined cataract and minimally invasive glaucoma surgery (MIGS): iStent | 3 |
|  |  |  |  |  |  |  |  |  |  |  |  |
| Sanchez-Sanchez 2021[43] | preperimetric glaucoma, glaucoma, AMD patients | AcrySof ReSTOR +3.00 (bifocal); AcrySof Panoptix (trifocal), Physiol FineVision (trifocal), and Tecnis Symfony (extended depth of focus)--> Healthy: 27.3% Bifocal Lens, 72.7% Trifocal Lens Preperimetric Glaucoma: 77.8% Bifocal Lens, 22.2% Trifocal Lens Glaucoma: 77.8% Bifocal Lens, 22.2% Trifocal Lens | Spain | 11 | 9 Preperimetric Glaucoma, 9 Perimetric Glaucoma | 67.64 (6.36) | Preperimetric Glaucoma: 68.33 (8.37) Glaucoma: 76.33 (6.61) | 45.5% M | Preperimetric Glaucoma: 55.6% M Glaucoma: 33.3% M | cataract surgery | 6 |
|  |  |  |  |  |  |  |  |  |  |  |  |
| Takai 2021[44] | OAG and regular corneal astigmatism exceeding 1.5 diopters (D) | AcrySof® IQ Toric IOL (Alcon Vision LLC, Fort Worth, TX, USA): 1 SN6AT3, 1 SN6AT4, 3 SN6AT5, 4 SN6AT6, 1 SN6AT7 | Japan | Non Toric IOL: 10 (10 eyes) | Toric IOL: 10 (10 eyes) | Non Toric: 76.9 ± 6.82 | Toric: 74.6 ± 7.20 | Non Toric: 6/4 | Toric: 3/7 | Combined Cataract Surgery and Microhook Ab Interno Trabeculotomy | 3 |
|  |  |  |  |  |  |  |  |  |  |  |  |
| Lopez Caballero 2022[45] | Phaco group: 13 mild, 17 moderate, 11 severe (38 OAG, 1 CAG, 2 PXF) Phaco + IStent: 11 mild, 7 moderate, 8 severe (18 OAG, 3 CAG, 2 PXF, 3 pigmentary) | AcrySof toric IOL | Spain | 31 (41 eyes) phaco | 21 (26 eyes) phaco +iStent | 72.87 ± 8.04 phaco | 71.67 ± 8.53 years phaco +iStent | 14/17 phaco | 13/8 phaco + iStent | trabecular micro bypass stents (iStent Inject) in patients with toric IOL (phacoemulsification) | 3 |
|  |  |  |  |  |  |  |  |  |  |  |  |
| Ichioka 2021[46] | Glaucomatous eyes with preexisting corneal astigmatism exceeding −1.5 D, Non Toric: 7 POAG, 2 EXG, 1 PACG Toric: 6 POAG, 4 EXG | non toric IOL (Vivinex iSert XY1, Hoya, Tokyo, Japan; n=10) or a toric IOL (Vivinex Toric XY1, Hoya; n=10) | Japan | Non Toric IOL: 10 (10 eyes) | Toric IOL: 10 (10 eyes) | Non Toric: 73.8±6.6 | Toric: 71.4±9.1 | Non Toric: 5/5 | Toric: 4/6 | non toric and toric IOLs during μLOT triple procedures | 3 |
|  |  |  |  |  |  |  |  |  |  |  |  |
| Brown 2015[47] | Glaucomatous eyes with corneal astigmatism: 79 OAG, 24 CACG, 20 Borderline Glaucoma/ OHT, 3 PXF | AcrySof toric IOL | US | NA | 87 (126 eyes) |  | 67.4 ± 8.4 |  |  | phacoemulsification |  |
|  |  |  |  |  |  |  |  |  |  |  |  |
| Ouchi 2015[22] | coexisting ocular pathologies | LENTIS MPlus LS-313MF30 and the LENTIS MPlus Toric LU-313MFT (Oculentis): 13/15 eyes | Japan | NA | 3 NTG, 1 Acute Glaucoma, 6 High Myopia, 2 Keratoconus, 2 BRVO, 2 Fundus Albipunctatus | NA | 51.4± 11.84 | NA | 4/7 | Phaco + IOL implantation | 6 |
|  |  |  |  |  |  |  |  |  |  |  |  |
| Kamath 2000[48] | Multifocal (81): 36 AMD, 11 Glaucoma, 6 Ocular HTN, 10 DR, 18 Others Monofocal (52): 14 AMD, 12 Glaucoma, 15 DR, 11 Others | Array® MFIOL | UK | 41 (52 eyes): monofocal AMO SI-40NB | 70 (81 eyes): 11 glaucoma + 6 ocular HTN patients had MFIOL, 12 glaucoma patients had monofocal IOL. | 72.4 years (range 45 to 91 years) | 74.5 years (range 48 to 87 years) | 12/29 | 26/44 | Phacoemulsification: 70 MFIOL, 48 Monofocal Phacoemulsification + trabeculectomy: 7 MFIOL, 3 Monofocal Phacoemulsification + pars plana vitrectomy: 1 MFIOL, 1 monofocal Extracapsular cataract extraction: 1 MFIOL, 0 Monofocal Secondary posterior chamber IOL (sulcus fixated): 2 MFIOL, 0 Monofocal | 4 |
|  |  |  |  |  |  |  |  |  |  |  |  |
|  |  |  |  |  |  |  |  |  |  |  |  |
| Rementería-Capelo 2022[49] | Glaucoma:6 (24%) Cornea guttata:4 (16%) Dry AMD: 3 12%)  Ocular HTN: 2 (8%) Amblyopia: 2 (8%) Corneal leukoma: 2 (8%) Epiretinal membrane: 1(4%) Macular telangiectasia: 1 (4%) Lagophthalmos due to facial nerve palsy: 1 (4%) Homonymous hemianopia: 1 (4%) previous LASIK surgery: 1 (4%) Daltonism: 1 (4%). | AcrySof IQ Vivity; Alcon | Spain | 25 patients (50 eyes) | 25 patients (50 eyes) | 71.36 years ± 5.61, range 60 to 81 | 71.84 ± 5.62 years, range 60 to 83 | 14/11 | 14/11 | Cataract surgery and iStent implantation | 3 |
| Kerr 2023[50] | EDOF group: 29 POAG eyes, 3 SOAG eyes Monofocal group: 23 POAG eyes, 3 SOAG eyes | EDOF (AcrySof IQ Vivity; Alcon), Monofocal (Clareon/SN6ATx/SN60WF; Alcon) IOLs | Australia | 13 patients (26 eyes) | 16 patients (32 eyes) | 78.4 ± 7.5 | 73.9 ± 5.0 | 3/10 | 8/8 | Cataract surgery and iStent/ Hydrus Stent implantation: 14 EDOF eyes, 10 monofocal eyes. Phacoemulsification: 26 EDOF eyes, 22 monofocal eyes | 3 |

Abbreviations: POAG: open angle glaucoma, SOAG: secondary open angle glaucoma, CAG: closed angle glaucoma, NTG: normal tension glaucoma, MIGS: Minimally Invasive Glaucoma Surgery, Pseudoexfoliation: PXF, HPA: (Hoddap Parrish Anderson) classification, Intraocular lens: IOL

**Table S3: Visual Results Table**

Includes visual outcome data such as visual acuity measures, contrast sensitivity and patient reported glare and spectacle independence.

| **Author/Year** | **1. Visual Field Parameters** | **2. Visual Acuity Outcomes (LogMAR)** | | | | **3. Visual Quality Outcomes** | | |
| --- | --- | --- | --- | --- | --- | --- | --- | --- |
|  |  | **Monocular UDVA, UIVA, UNVA** | **Monocular CDVA** | **Binocular UDVA, UIVA, UNVA** | **Binocular CDVA** | **Glares and Haloes** | **Patient Satisfaction** | **Spectacle Independence** |
| Ferguson 2023[40] | 1. Mean Deviation: −1.4 ± 1.9 dB 2. Pattern Deviation: 2.1 ± 1.1 3. Visual Field Index: 97.6 ± 2.4 | Monocular UDVA:  Monocular UIVA: 0.18 ± 0.12 Monocular UNVA: 0.31 ± 0.18 |  | Binocular UDVA: 0.03 ± 0.12 Binocular UIVA: 0.17 ± 0.12 Binocular UNVA: 0.31 ± 0.17 | −0.06 ± 0.07 | mean response of 2.6 ± 1.3 (scale of 1-5) | 85% will choose same IOL | For Near Distance: 38% (n=10) For Intermediate Distance: 50% (n=13) For Far Distance: 92% (n=24) |
|  |  |  |  |  |  |  |  |  |
| Bissen-Miyajima 2023[41] | Reported Individually |  |  |  | mean DCVAs at the distances of 5, 1, and 0.5 m were −0.15, −0.05, and 0.06 logMAR | 4 None, 5 Mild, 1 Modest |  |  |
|  |  |  |  |  |  |  |  |  |
| Ichioka 2022[42] |  | UCVA: Non toric: 0.45 ± 0.31, Toric: 0.14 ± 0.15 |  |  | BCVA: Non toric: 0.04 ± 0.06, Toric: 0.02 ± 0.06 |  |  |  |
|  |  |  |  |  |  |  |  |  |
| Sanchez-Sanchez 2021[43] |  | Monocular UDVA:  Healthy: 0.00 (0.09) Preperimetric Glaucoma: 0.13 (0.21) Glaucoma: 0.32 (0.35) | Healthy: −0.03 (0.04)  Preperimetric Glaucoma: 0.02 (0.05) Glaucoma: 0.18 (0.25) |  |  |  | Vision Satisfaction: Healthy: 3.50 (0.71) Glaucoma: 2.89 (0.33) Preperimetric glaucoma: 3.22 (0.67) | For Near Distance: 56% For Intermediate Distance: 89% For Far Distance: 68% |
| Takai 2021[44] |  |  |  | UCVA Toric: 0.23 ± 0.25 Non Toric: 0.45 ± 0.26 | BCVA Toric: −0.11 ± 0.08 Non Toric: 0.05 ± 0.12 |  |  |  |
|  |  |  |  |  |  |  |  |  |
| Lopez Caballero 2022[45] | Mean Deviation:  Phaco: − 7.31 (4.21) − 17.29 to − 1.35 Phaco + iStent: − 7.40 (7.84) − 29.44 to − 1.08 |  |  | UCVA Phaco group: 0.04 (0.07) Phaco + IStent:0.03 (0.11) | Phaco group: 0.02 (0.06) Phaco + IStent: 0.01 (0.04) |  |  |  |
|  |  |  |  |  |  |  |  |  |
| Ichioka 2021[46] |  |  |  | UCVA  Non Toric: 0.33±0.30 Toric: 0.07±0.07 | Post op BCVA:  Non Toric: 0.02±0.06 Toric: −0.01±0.09 |  |  |  |
|  |  |  |  |  |  |  |  |  |
|  |  |  |  |  |  |  |  |  |
| Brown 2015 [47] | Mean Deviation preoperative VF loss: -4.00 ± 4.91 | Monocular UDVA: 0.04 ± 0.08 | 0.01 ± 0.03 |  | 0.01 ± 0.03 logMAR (20/20.5 Snellen) |  |  |  |
|  |  |  |  |  |  |  |  |  |
| Ouchi 2015[22] |  |  |  | Binocular UDVA: -0.001±0.11 Binocular UNVA: 0.2 ± 0.19 | −0.07±0.11 |  |  | For Distance: all 11 eyes independent For Near: 9 eyes independent, 1 needed glasses occasionally, 1 needed glasses constantly |
|  |  |  |  |  |  |  |  |  |
| Kamath 2000 [48] |  |  |  | UCVA >6/12: 29 UCVA >= N8: 82 UCVA >-6/12 and N8: 24 | BCVA >6/12: 94 BCVA >= N8: 88 BCVA >-6/12 and N8: 88 |  |  |  |
|  |  |  |  |  |  |  |  |  |
| Rementería-Capelo 2022[49] |  | Control: −0.01 ± 0.07, 0.30 to −0.20  Study: 0.03 ± 0.08, 0.30 to −0.10 | Control: −0.01 ± 0.06, 0.20 to −0.20  Study: 0.00 ± 0.06, 0.20 to −0.10 | Control: −0.06 ± 0.06, 0.00 to −0.20 Study: −0.05 ± 0.06, 0.10 to −0.20 | Control: −0.06 ± 0.06, 0.00 to −0.20 Study: −0.06 ± 0.06, 0.00 to −0.20 | Control: 28% halos, 48% glare  Ocular Pathology group: 60% halos, 56% glare | Satisfaction on vision scale 1-5: Control 3.52 ± 0.51, Study answers 3.84 ± 0.37. 52% of the healthy group and 84% of the study group reported being “very satisfied” and all patients reported being “fairly/very satisfied.” | For distance: All spectacle independent  For Intermediate: 2 controls (8%) needed spectacles  For near: 3 controls (12%) needed spectacles, 12 controls (48%) sometimes, 10 (40%) never  1 patient (4%) always required, 14 patients (56%) sometimes, 10 patients (40%) never |
|  |  |  |  |  |  |  |  |  |
| Kerr 2023 [50] | Mean deviation (dB): −1.6 ± 2.4 Visual field pattern SD: 2.7 ± 1.8 |  |  | For EDOF:  UDVA: 0.12 ± 0.15  UIVA: 0.06± 0.16  UNVA: 0.29± 0.10  For Monofocal:  UDVA: 0.10 ± 0.13  UIVA: 0.39± 0.10  UNVA: 0.55± 0.18 | For EDOF: 0.01 ± 0.09 For Monofocal: 0.00 ± 0.10 | For EDOF: 9 Not at all, 3 A little, 2 Somewhat, 2 Very For Monofocal: 7 not at all, 4 a little, 1 somewhat, 1 very | For EDOF:  For Distance: All 16 very satisfied  For Intermediate: All 16 very satisfied  For Near: 12 very satisfied, 4 somewhat satisfied  For Monofocal:  For Distance: 9 very satisfied, 4 somewhat satisfied  For Intermediate: 6 very satisfied, 6 somewhat satisfied, 1 somewhat dissatisfied  For Near: 5 very satisfied, 4 somewhat satisfied, 2 somewhat dissatisfied, 2 very dissatisfied | For EDOF: For Distance: 13 Never needed, 3 Rarely  For Intermediate: 13 never needed, 3 rarely  For Near: 7 never needed, 4 rarely, 2 sometimes, 2 often, 1 always    For Monofocal:  For distance: 8 never needed, 1 rarely, 1 sometimes, 3 always  For intermediate: 5 never, 1 sometimes, 3 often, 4 always  For ner: 1 sometimes, 1 often, 11 always |
|  |  |  |  |  |  |  |  |  |

Abbreviations: POAG: open angle glaucoma, SOAG: secondary open angle glaucoma, CAG: closed angle glaucoma, NTG: normal tension glaucoma, MIGS: Minimally Invasive Glaucoma Surgery, Pseudoexfoliation: PXF, HPA: (Hoddap Parrish Anderson) classification, Intraocular lens: IOL

UDVA: uncorrected distance visual acuity, UIVA: uncorrected intermediate visual acuity, UNVA: uncorrected near visual acuity, CDVA: corrected distance visual acuity,

MRSE: mean spherical refractive equivalent

**Table S4: Refractive Outcomes Table**

Provides preoperative and postoperative clinical characteristics [MRSE, Astigmatism, Corneal Astigmatism, IOP, OP Medications, Axial Length]

| **Author/Year** | **4. Refractive Outcomes (D)** | | | | | | | | | **5. IOP and Glaucoma Medication Outcomes** | | | | | **6.Others** | |
| --- | --- | --- | --- | --- | --- | --- | --- | --- | --- | --- | --- | --- | --- | --- | --- | --- |
|  | **Pre Op MRSE** | **Post Op Mean MRSE** | **Changes in Refractive Spherical Error** | **Pre Op Astigmatism** | **Post Op Astigmatism** | **Change in Astigmatism** | **Pre Op corneal astigmatism** | **Post Op corneal astigmatism** | **Change in corneal astigmatism** | **Pre Op IOP (mmHg)** | **Post Op IOP (mmHg)** | **Changes in IOP (mmHg)** | **Pre Op Medications** | **Post Op Medications** | **Change in medications** | **Axial Length (mm)** |
| Ferguson 2023 [40] |  | −0.27 ± 0.35 D |  |  |  |  | Non toric: -2.60 ± 0.94, Toric-2.12 ± 0.43 | Non toric: -2.13 ± 1.09, Toric-1.72 ± 0.71 | Non toric: 0.47 ± 0.25, Toric:0.40 ± 0.25 | 18.5 ± 4.6 | 13.3 ± 2.9 | All eyes: - >5 mmHg from baseline |  |  |  |  |
|  |  |  |  |  |  |  |  |  |  |  |  |  |  |  |  |  |
| Bissen-Miyajima 2023 [41] |  | reported individually |  |  |  |  |  |  |  | 12 (2.5) | reported individually |  |  |  |  | 25.9 (2.0) |
|  |  |  |  |  |  |  |  |  |  |  |  |  |  |  |  |  |
| Ichioka 2022 [42] | Non toric:1.11 ± 0.91, Toric: 1.33 ± 1.85 | Non toric:1.03 ± 0.75, Toric: 0.19 ± 0.56 | Non toric-0.08 ± 0.35, Toric:-1.14 ± 0.62 | Non toric-2.25 ± 0.68, Toric-2.39 ± 0.50 | Non toric: -2.03 ± 0.63, Toric: -0.67 ± 0.53 | Non toric: 0.22 ± 0.32, Toric:+ 1.72 ± 0.25 | Non toric: -2.60±0.94, Toric: -2.12±0.43 | Non toric: -2.13±1.09, Toric: -1.72±0.71 | Non toric: 0.47±0.25, Toric: 0.40±0.25 | Non Toric: 14.8 ± 4.0, Toric: 15.0 ± 3.7 | Non Toric:12.1 ± 3.7, Toric: 13.6 ± 1.7 | Non Toric:-2.7 ± 1.8, Toric: -1.4 ± 1.0 | Non Toric: 2.3 ± 1.9, Toric: 2.2 ± 1.2 | Non Toric: 1.6 ± 0.9, Toric: 1.2 ± 1.1 | Non Toric: -0.8 ± 0.5, Toric: -1.0 ± 0.5 |  |
|  |  |  |  |  |  |  |  |  |  |  |  |  |  |  |  |  |
| Sanchez-Sanchez 2021[43] |  |  |  |  |  |  |  |  |  |  |  |  |  |  |  |  |
| Takai 2021[44] |  | Toric: (1.30 ± 0.68 D) Non Toric:(2.25 ± 0.62 D) |  | Toric: 2.70 ± 0.90 , Non Toric: 2.08 ± 0.58 | Toric: 1.30 ± 0.68 , Non Toric: 2.25 ± 0.62 |  | Toric: 2.09 ± 0.50, Non toric: 1.75 ± 0.30 |  |  | Toric: 19.5 ± 6.17 Non Toric:18.8 ± 3.55 | Toric: 12.4 ± 3.41  Non Toric:13.0 ± 1.70 | Toric: 33.9 ± 15.6, Non Toric:29.4 ± 11.7 | Toric: 2.70 ± 1.06 Non Toric:2.70 ± 0.80 | Toric: 1.70 ± 0.95 Non Toric: 2.10 ± 0.88 |  |  |
|  |  |  |  |  |  |  |  |  |  |  |  |  |  |  |  |  |
| Lopez Caballero 2022[45] |  | Phaco: − 0.18 (0.52) −2.00 to+0.50 Phaco + iStent: − 0.23 (0.42) − 1.25–0.00 |  |  | Phaco: − 0.26 (0.40) Phaco + iStent: − 0.11 (0.28) |  | Biometric Corneal Astigmatism Phaco: 1.55 (0.74) 0.60–4.16 Phaco + iStent: 1.35 (0.71) 0.57–3.50 |  |  | Phaco:  15.63 (3.23)   Phaco + istent: 17.19 (2.99) | Phaco: 14.00 (3.11) Phaco + istent: 14.16 (3.26) | Phaco: 0.008 Phaco + iStent: < 0.001 | Phaco: 1.63 (0.80) Phaco + istent: 2.12 (0.65) | Phaco: 1.34 (0.91) Phaco + istent: 0.44 (0.71) | Phaco: 0.022 Phaco + iStent: < 0.001 | Phaco: 24.35 (1.50) 22.38–28.79 Phaco + iStent: 24.19 (1.13) 22.19–26.57 |
|  |  |  |  |  |  |  |  |  |  |  |  |  |  |  |  |  |
| Ichioka 2021 [46] | Non Toric:−0.15 ± 1.4 Toric: −0.78 ± 3.95 | Post Op refractive spherical error  Non Toric: +0.40±0.44 Toric: +0.57±0.18 | Non Toric:+0.55 ± 0.46 Toric: −0.78 ± 1.13 | Non Toric: −1.98 ± 1.33 Toric: −2.08 ± 1.19 | Non Toric: −1.53±0.74 Toric: −0.63±0.56 | Non Toric:+0.45 ± 0.45 Toric: +1.45 ± 0.46 | Non Toric:−2.08 ± 0.72 Toric: −2.37 ± 0.73 | Non Toric: −1.49 ± 0.78 Toric: −2.27 ± 1.08 | Non Toric: +0.59 ± 0.36 Toric: +0.10 ± 0.21 | Non Toric: 15.8±2.5 Toric: 16.7±5.1 | Non Toric: 12.4±2.3 Toric:13.0±2.8 | Non Toric: −3.4±0.9 Toric: −3.7±1.5 | Non Toric: 3.0±0.9 Toric: 2.9±1.1 | Non Toric: 2.4±0.8 Toric: 2.1±0.7 | Nontoric:−0.6±0.2 Toric: −0.8±0.4 |  |
|  |  |  |  |  |  |  |  |  |  |  |  |  |  |  |  |  |
|  |  |  |  |  |  |  |  |  |  |  |  |  |  |  |  |  |
| Brown 2015 [47] | -2.11 ± 3.70 | -0.28 ± 0.44 |  | 1.47 ± 1.10 | 0.31 ± 0.37 |  |  |  |  | 17.1 ± 3.3 | 14.8 ± 2.6 | mean -2.3mmHg in all toric groups |  |  |  | 24.13 ± 1.61 |
|  |  |  |  |  |  |  |  |  |  |  |  |  |  |  |  |  |
| Ouchi 2015 [22] |  | − 0.11 ± 0.54 D (−0.75 to 0.625 D) |  | -9.48 ± 6.64 D | manifest cylinder was 0.36±0.66 D (0 to 1.75 D); |  | 1.38 ± 0.55 D | 1.54 ± 0.53 D (0.75 to 2.5 D) |  |  |  |  |  |  |  | pre: 26.5 ± 2.55 mm, mean photopic pupil diameter is 3.82 ± 0.61 (2.70–4.62) mm |
|  |  |  |  |  |  |  |  |  |  |  |  |  |  |  |  |  |
| Kamath 2000 [48] |  |  |  |  |  | MFIOL Eyes Sphere <+/- 1.0: 4 Sphere >+/- -1.0: 19 Sphere >+/- +1.0: 3 Cylinder >1/50 and Sphere <+/- 1.00: 4 Cylinder >1/50 and Sphere >1.00: 5 |  |  |  |  |  |  |  |  |  |  |
|  |  |  |  |  |  |  |  |  |  |  |  |  |  |  |  |  |
| Rementería-Capelo 2022 [49] |  | Healthy: −0.02 ± 0.08 (−0.50 to 0.00 )   Ocular Pathology: −0.06 ± 0.16 (−0.75 to 0.00) |  |  | Healthy: −0.06 ± 0.26 (−1.75 to 0.00)   Ocular Pathology: −0.04 ± 0.16 (−0–75 to 0.00) |  |  |  |  |  |  |  |  |  |  | Control: 24.01 ± 0.79, 22.58 to 26.43 Study: 23.96 ± 1.11, 21.15 to 26.67 |
|  |  |  |  |  |  |  |  |  |  |  |  |  |  |  |  |  |
| Kerr 2023 [50] | For EDOF: 1.10 ± 1.97 For monofocal: 0.19 ± 2.32 | For EDOF: −0.35 ± 0.34  For monofocal: −0.36 ± 0.42 |  | For EDOF: −0.69 ± 0.54 For monofocal: −0.83 ± 0.72 |  |  |  |  |  | 15.2 ± 4.4 |  |  | 0.5 ± 0.8 |  |  | 23.40 ± 0.88 |
|  |  |  |  |  |  |  |  |  |  |  |  |  |  |  |  |  |
